# Gastrointestinal presentation of Kawasaki disease: A red flag for severe disease?

**DOI:** 10.1371/journal.pone.0202658

**Published:** 2018-09-04

**Authors:** Marianna Fabi, Elena Corinaldesi, Luca Pierantoni, Elisa Mazzoni, Chiara Landini, Barbara Bigucci, Gina Ancora, Laura Malaigia, Tetyana Bodnar, Giorgia Di Fazzio, Francesca Lami, Enrico Valletta, Cristina Cicero, Giacomo Biasucci, Lorenzo Iughetti, Federico Marchetti, Paola Sogno Valin, Sergio Amarri, Sandra Brusa, Monica Sprocati, Giuseppe Maggiore, Ada Dormi, Paolo Lanzoni, Andrea Donti, Marcello Lanari

**Affiliations:** 1 Pediatric Cardiology and Adult Congenital Unit, University of Bologna, S.Orsola-Malpighi Hospital, Bologna, Italy; 2 Department of Pediatrics, Ramazzini Hospital, Carpi, Italy; 3 Department of Pediatrics, University of Bologna, S.Orsola-Malpighi Hospital, Bologna, Italy; 4 Department of Pediatrics, Maggiore Hospital, Bologna, Italy; 5 Department of Pediatrics, Infermi Hospital, Rimini, Italy; 6 Department of Pediatrics, Bufalini Hospital, Cesena, Italy; 7 Department of Pediatrics, Arcispedale Santa Maria Nuova, IRCCS, Reggio Emilia, Italy; 8 Department of Pediatric, Department of Medical and Surgical Sciences for Mothers, Children and Adults, University of Modena and Reggio Emilia, Reggio Emilia, Italy; 9 Department of Pediatrics, G.B.Morgagni–L. Pierantoni Hospital, AUSL della Romagna, Forlì, Italy; 10 Department of Pediatrics, Guglielmo da Saliceto Hospital, AUSL, Piacenza, Italy; 11 Department of Pediatrics, Santa Maria delle Croci Hospital, AUSL della Romagna, Ravenna, Italy; 12 Department of Pediatrics, Santa Maria della Scaletta Hospital, Imola, Italy; 13 Department of Pediatrics, Arcispedale Sant’Anna, Ferrara, Italy; 14 Department of Medical and Surgical Sciences DIMEC, University of Bologna, Bologna, Italy; Cedars Sinai Medical Center, UNITED STATES

## Abstract

**Background:**

Kawasaki disease (KD) is a febrile systemic vasculitis of unknown etiology and the main cause of acquired heart disease among children in the developed world. To date, abdominal involvement at presentation is not recognized as a risk factor for a more severe form of the disease.

**Objective:**

To evaluate whether presenting abdominal manifestations identify a group at major risk for Intravenous immunoglobulin (IVIG)-resistance and coronary lesions.

**Methods:**

Retrospective study of KD patients diagnosed between 2000 and 2015 in 13 pediatric units in Italy. Patients were divided into 2 groups according to the presence or absence of abdominal manifestations at onset. We compared their demographic and clinical data, IVIG-responsiveness, coronary ectasia/aneurysms, laboratory findings from the acute and subacute phases.

**Results:**

302 patients (181 boys) were enrolled: 106 patients with, and 196 patients without presenting abdominal features. Seasonality was different between the groups (p = 0.034). Patients with abdominal manifestations were younger (p = 0.006) and more frequently underwent delayed treatment (p = 0.014). In the acute phase, patients with abdominal presentation had higher platelet counts (PLT) (p = 0.042) and lower albuminemia (p = 0.009), while, in the subacute phase, they had higher white blood cell counts (WBC) and PLT (p = 0.002 and p < 0.005, respectively) and lower red blood cell counts (RBC) and hemoglobin (Hb) (p = 0.031 and p 0.009). Moreover, the above mentioned group was more likely to be IVIG-resistant (p < 0.005) and have coronary aneurysms (p = 0.007). In the multivariate analysis, presenting abdominal manifestations, age younger than 6 months, IVIG- resistance, delayed treatment and albumin concentration in the acute phase were independent risk factors for coronary aneurysms (respectively p<0.005, <0.005, = 0.005 and 0.009).

**Conclusions:**

This is the first multicenter report demonstrating that presenting gastrointestinal features in KD identify patients at higher risk for IVIG-resistance and for the development of coronary aneurysms in a predominantly Caucasian population.

**Clinical trial registration:**

8/20014/O/OssN.

## Introduction

Kawasaki disease (KD) is a febrile systemic vasculitis of unknown etiology which usually affects children younger than 5 years of age, and it is the main cause of acquired heart disease in the developed world. It mainly affects small and medium-sized arteries, leading to coronary artery lesions (CALs) in up to 25% of untreated cases [1]. Prompt identification and adequate treatment reduce the incidence of CALs to approximately 3%, reducing the risk of sudden death and myocardial ischemia in childhood and adulthood. Although conflicting data exist regarding the risk of CALs in patients with various clinical presentations [2,3], incomplete [2, 4] and atypical forms of KD [5] seem to be related to a higher risk of coronary involvement [6]. Moreover, many studies have demonstrated that failure to respond to the initial treatment with intravenous immunoglobulin (IVIG) increases the risk of developing coronary anomalies and giant aneurysms [7, 8]. Vasculitic changes can also occur in peripheral and visceral arteries (e.g. cerebrovascular, renal and gastrointestinal systems). Digestive tract involvement is reported in approximately 20–35% of cases [1, 9, 10, 11, 12] with different clinical manifestations (vomiting, diarrhea, abdominal pain, abdominal distension, jaundice, paralytic ileus, hepatomegaly, hydrops of gallbladder, and, much less frequently, pancreatitis, gastrointestinal obstruction/ pseudo-obstruction) and echographic findings. Gastrointestinal symptoms at KD onset can complicate clinical recognition [12, 13, 14], lead to unnecessary surgical interventions and cause therapeutic delay, thus increasing the risk of CALs. To date, no multicenter study has investigated whether the presence of clinical abdominal involvement is a marker of more severe disease. The aim of our study was to evaluate whether gastrointestinal symptoms at presentation, regardless of their magnitude, can identify a group at higher risk for IVIG-resistance and CALs.

## Patients and methods

The multicenter retrospective study included all the patients diagnosed with KD at 13 pediatric units in Emilia-Romagna, a region in the north of Italy, between 2000 and 2015. Every diagnosis of KD was made in accordance with the 2004 American Heart Association Recommendations[1], distinguishing complete and incomplete/atypical forms. Disease onset was defined as the first day of fever. In accordance with the 2004 American Heart Association Recommendations, patients were treated with IVIG at 2 g/kg in a single infusion before the tenth day of fever, together with aspirin at 80–100 mg/kg/day, which was then switched to 3–5 mg/kg/day once the patient was afebrile. Intravenous immunoglobulin-resistance was defined as persistent/recrudescent fever at least 36 hours, but not longer than 7 days, after the completion of the first IVIG infusion. Treatment was defined as late when the first dose of IVIG was administered after the 10th day of fever. Echocardiography was performed at each participating center at diagnosis and between the 11th and 20th day after diagnosis. Coronary artery abnormalities were classified as ectasia and aneurysms by the local cardiac sonographer according to the morphology and measurements of the internal diameters of the coronary arteries (right, left anterior descending and circumflex coronary arteries). Coronary artery lesions were diagnosed according to the Japanese Ministry of Health and Welfare criteria as the maximal luminal diameter greater than 3 mm in children younger than 5 years and greater than 4 mm in children older than 5 years or a diameter 1.5 times the size of the surrounding segment or a clearly irregular lumen [15]. A database was prospectively created and then retrospectively reviewed; it included demographic features and clinical characteristics, days of fever before IVIG treatment, total days of fever, IVIG-responsiveness, laboratory values from the acute (from onset to 10th day) and subacute (11th to 20th day after onset) stages of the illness (white blood cell count [WBC], neutrophil and lymphocyte percentage, red blood cell count [RBC], hemoglobin [Hb], platelets [PLT], C-reactive protein [CRP], erythrocyte sedimentation rate [ESR], serum albumin, alanine aminotransferase [ALT], gamma-glutamyl transferase [gamma GT], sodium [Na]). Erythrocyte sedimentation rate was only performed before IVIG infusion. The gastrointestinal complaints were all registered during the acute stage of the illness using standard definitions. The following gastrointestinal manifestations were considered: vomiting, diarrhea, abdominal pain, abdominal distension, paralytic ileus, jaundice, pancreatitis and pseudo-obstruction. The presence of vomiting and diarrhea was documented based on standard definition if reported by caregivers and/or directly observed during the acute phase of the hospital stay. Abdominal pain was defined on physical examination using a pain assessment scale appropriate for age [the Face, Legs, Activity, Cry, Consolability scale (FLACC scale), Wong Baker FACES pain rating scale]. Paralytic ileus, obstruction, jaundice and pancreatitis were clinically suspected and confirmed by imaging and laboratory findings, when appropriate. We excluded patients with positive fecal cultures, anatomical malformations and etiologies other than KD from the data analysis.

The study was approved by the Health Research Ethics Committee of the Sant’Orsola Malpighi University Hospital (approval number 8/20014/O/OssN).

For analysis we divided the KD patients into 2 groups according to the presence or absence of any abdominal manifestation at presentation.

We subsequently compared demographic and clinical characteristics, the presence of CALs and laboratory findings from the acute and subacute phases between the 2 groups. For categorical variables, the percentage of patients in each category was calculated and compared with Chi square or Fisher’s exact test, when appropriate. Continuous data are presented as mean±standard deviation (SD). Non-parametric data were compared using the Mann-Whitney U-test, and parametric data using the t-test.

To assess whether presenting abdominal manifestations had a significant effect on the outcome of coronary artery involvement, we conducted a multivariate analysis using coronary aneurysms as the outcome variable and we included the presence of abdominal manifestations, IVIG-responsiveness, late treatment, non-coronary involvement (aortic and mitral regurgitation, ventricular dysfunction and pericarditis), sex, age at onset, age younger than 6 months, WBC, PLT, albumin, sodium and CRP of the acute phase as possible independent risk factors. To assess parametric variables, we used binary logistic regression; to assess nonparametric dichotomous variables we used contingency tables and Fisher’s test; to assess nonparametric non dichotomous variables we used regression analysis with Dummy variables.

The level of statistical significance was set at p < 0.05.

The analysis for this study was performed with an SPSS Statistics V23 for Windows.

## Results

A total of 302 consecutive patients diagnosed with KD were enrolled in the study. One hundred and six patients had gastrointestinal manifestations at disease onset and 196 did not. Patients with abdominal involvement were statistically significantly younger (median age, number of patients younger than 1 year and younger than 6 months), had different seasonality of KD (p = 0.034), were more frequently treated late (p = 0.014), were IVIG-resistant (p < 0.005) and developed more coronary aneurysms (p = 0.007) (see Table 1).

**Table 1:** Demographics, clinical characteristics, IVIG-responsiveness, delayed treatment and coronary ectasia and aneurysms of patients with Kawasaki disease presenting with and without abdominal symptoms.

	No abdominal manifestations (n = 196)	Abdominal manifestations (n = 106)	P
Male, no.(%)	118 (64.9)	63 (35.1)	n/s.
Age at onset (months), median (±SD)	38.8 (±31.6)	28.4 (±31.7)	**0.006**
Age at onset<6 months, n(%)	15 (7.6)	20 (18.8)	**<0.005**
Age at onset<1 year, n(%)	27 (13.8)	35 (33%)	**0.004**
Season			**0.034**
Winter, n(%)	48 (24.5)	43(40.6)	
Spring, n(%)	56 (28.6)	25 (23.6)	
Summer, n(%)	36 (18.3)	13 (12.2)	
Autumn, n (%)	56 (28.6)	25 (23.6)	
Race			n/s.
Caucasian, n(%)	177 (90.3)	94(88.6)	
Asian, n(%)	10 (5.1)	6 (5.6)	
Hispanic, n(%)	2 (1)	1 (0.9)	
African, n(%)	2 (1)	2 (1.8)	
Clinical criteria			n/s.
Typical, n(%)	140 (71.5)	76 (71.7)	
Atypical/incomplete, n(%)	56 (28.5)	30 (28.3)	
Total days of fever: mean, ±SD	7.8±4;169	10±7; 84	**0.012**
Responder, n(%)	157 (80.1)	62 (58.4)	**<0.005**
IVIG Resistant, n(%)	18 (9.2)	21 (19.8)	**<0.005**
Delayed treated, n(%)	13 (6.6)	17 (16)	**0.014**
Not treated, n(%)	8 (4.1)	6 (5.7)	n/s.
Coronary ectasia, n(%)	33 (16.8)	17 (16)	n/s.
Coronary aneurysms, n(%)	4 (2)	10 (9.4)	**0.007**

n, number; SD, standard deviation; IVIG, Intravenous immunoglobulin; n/s, non-significant

Patients with abdominal symptoms had significantly lower serum albumin levels (p = 0.009) and higher PLT (p = 0.042) in the acute stage, while the had higher WBC (p < 0.005) and PLT (p < 0.005), and lower RBC and Hb (p = 0.031 and 0.009, respectively) in the subacute stage (see Table 2).

**Table 2:** Laboratory values of the acute and subacute phase of patients with Kawasaki disease presenting with and without abdominal symptoms.

	No abdominal manifestations	Abdominal manifestations	P
A-WBC (10^3^/mm^3^)	15.2±5.8	15.6±5.6	n/s.
A-N(%)	66.8±15.3	65.1±14	n/s.
A-L(%)	22.4±12.8	24.7±12.3	n/s.
A-RBC (10^3^/mm^3^)	4250±543	4133±831	n/s
A-Hb (g/dl)	11.±1.2	11±1.1	n/s
A-PLT (10^3^/mm^3^)	390±176	434±179	**0.042**
A-CRP (mg/dl)	12.3±8.5	12.9±10	n/s
A-ESR (mm/h)	68±31.5	61.7±32.7	n/s.
A-ALT (IU/l)	76±123	91±129	n/s
A-gammaGT (IU/l)	65±86	62.8±78	n/s.
A-Na (MEq/l)	135±3.6	134.9±3.4	n/s
A-albuminemia (g/dl)	3.5±0.7	3.2±0.6	**0.009**
S-WBC (10^3^/mm^3^)	10.8±4.8	15.9±5.7	**<0.005**
S-N(%)	41.4±16.7	40±17.3	n/s
S-L(%)	45.5±16.7	47.±17.2	n/s
S-RBC (10^3^/mm^3^)	4231.7±537	4082.4±510	**0.031**
S-Hb (g/dl)	10.8±1.4	10.3±1.5	**0.009**
S-PLT (10^3^/mm^3^)	615±224.8	739±272	**<0.005**
S-CRP (mg/dl)	3±3.9	2.2±3.4	n/s

A-, acute phase, S-, subacute phase; WBC, white blood cells; N, neutrophils; L, lymphocytes; RBC, red blood cells; Hb stands for hemoglobin; PLT, platelets; CRP, C-reactive protein; ESR, erythrocytes sedimentation rate; ALT, alanine aminotransferase; gamma GT, gamma-glutamyl transferase; Na, Sodium.

In our cohort, children younger than 6 months compared to others were more likely to present abdominal symptoms (58.1% versus 32.1%, p = 0.004) and to develop coronary aneurysms (16.1% versus 3.3%, p = 0.001). In particular, in a group of children younger than 6 months, those with abdominal symptoms had a higher incidence of aneurysms (5/20 versus 0/15, p = 0.048) compared to those without.

To assess which factor had the most significant effect on coronary outcome, we conducted a multivariate analysis using coronary aneurysms as the outcome variable. We included the presence of abdominal manifestations, sex, age at onset (months, median±SD), age younger than 6 months, IVIG-responsiveness, late treatment, non-coronary artery involvement (aortic and mitral regurgitation, ventricular dysfunction and pericarditis), WBC, PLT, albumin, sodium and CRP from the acute phase in multivariate analysis. Multivariate analysis identified presenting abdominal manifestations, age younger than 6 months, IVIG-unresponsiveness, delayed treatment and lower albuminemia as independent risk factors for the development of coronary aneurysms (see Table 3).

**Table 3:** Multivariate analysis with coronary aneurysms as the outcome variable.

	OR	95% confidence interval	P
Abdominal manifestations	5	1.55–16.6	<0.005
Age <6 months	6.1	1.3–28.5	0.021
IVIG-unresponsiveness	6.7	1.8–24.5	<0.005
Late treatment with IVIG	7	1.78–27.9	0.005
A-albuminemia	0.25	0.09–0.7	0.009

A-albumin, albumin level of the acute phase of KD; IVIG, Intravenous Immunoglobulin; OR, Odd Ratio

## Discussion

Although mild abdominal complaints can frequently occur in KD [1, 5], their presence has not been statistically associated with more severe disease to date.

To our knowledge, this is the first multicenter report demonstrating that clinical abdominal manifestations at KD onset, regardless of their magnitude, are a marker for more severe disease, identifying patients at higher risk for IVIG-unresponsiveness and coronary aneurysms in our population.

Our findings show that gastrointestinal presentation, age younger than 6 months, resistance to conventional treatment, late treatment, and low albuminemia are risk factors for severe coronary lesions. Recently, Salgado et al. demonstrated that infants younger than 6 months have poor coronary outcomes despite timely diagnosis and adequate treatment and a similar prevalence of IVIG-resistance compared to older children [16]. Our data not only show that children under 6 months of age presented abdominal symptoms and coronary aneurysms more often, but also that in this age-group coronary aneurysms were more frequent when abdominal complaints were present.

It is worthy to note that while it is well known that younger age, lower albumin levels, late treatment and IVIG-unresponsiveness are risk factors for a more severe form of the disease, our data demonstrate that presenting abdominal manifestations represent an independent risk factor for coronary aneurysms in our mostly Caucasian cohort.

Moreover, patients with these presenting symptoms are more likely to be diagnosed during the winter and to be treated late.

The mechanism of gastrointestinal involvement has not yet been clarified. An autoptic study performed in patients with KD found systemic vasculitis and inflammatory lesions in various organs and tissues, including the heart and digestive system (sialoduct-adenitis, catarrhal enteritis, hepatitis, cholangitis, hydrops of gallbladder, pancreatitis, pancreas adenitis). The authors propose that vasculitis of a certain system can cause the corresponding clinical manifestations [17]. Increased intestinal permeability with capillary leak and hypoalbuminemia can result from different mechanisms mediated by hormones, such as thyroid hormones and glucocorticoids, nerves, and cytokines, such as interleukin-1, 2, 6 and interferon alpha[18]. Of note, a selective expansion of circulating Vβ2T cells has been found in the small intestinal mucosa and in the blood, presumably caused by exotoxines produced by bacteria colonizing jejunal mucosa in these patients [19]. Moreover, as recently demonstrated, intestinal microbiota [20, 21] plays a crucial role in the development of KD arteritis in a mouse model.

Rowley et al. previously demonstrated that IgA plasma cells infiltrate the vascular wall in children with KD [22]. Since maturation of the secretory IgA response occurs in young infants in whom the systemic IgA is immature, Rowley hypothesized that IgA response in KD could be the result of the stimulation by an antigen entering at a mucosal site, and then demonstrated increased IgA plasma cells in 14/14 (100%) of patients with KD in the trachea, pancreas, kidney and coronary artery and in 7/12 (58.3%) patients with KD in tissue around small bile ducts, and in both children with KD and of age-matched controls in the gastrointestinal tract [23]. These findings could suggest that the causative agent enters through respiratory or gastrointestinal tract where they activate an immune response. Increased gut permeability is also supported by the findings of Ohshio et al. who demonstrated high levels of secretory IgA in the acute stage and IgA-circulating immune complex in the subacute stage of the disease. [24]. More recentely Takeshita et al. showed high titers of serum level of anti-lipid A IgA, especially IgA2-subclass, in acute and subacute phase. Since Ig A2 levels are used as markers of active mucosal events, inflammation and permeability of intestinal mucosa is one option where the immune response could take place in KD [25]. Moreover, starting from the observation of high prevalence of celiac disease in patients with KD compared to non-KD controls, Stagi et al. speculated that involvement of intestinal mucosa in KD can increase permeability and participate in inducing celiac disease [26].

Yi et al.[12] reported that gallbladder distension during the acute stage of KD is significantly correlated with coronary artery complications. Eladawy et al. [27] documented a correlation between abnormal liver function and IVIG-unresponsiveness, but failed to prove an association with CALs. In our population, aspartate aminotransferase (AST) and gamma GT levels were not significantly different between the 2 groups.

A review of 18 reports of KD and abdominal surgical complications by Yaniv et al. found a male/female ratio of 2.4:1 with an average age of 35.8±44.4 months and, importantly, that 55% of these patients had cardiac involvement, mostly coronary aneurysms, which had resolved at follow-up in about 85% of cases [10]. Zulian et al. [28] reported 10 cases of abdominal surgical-onset KD for patients who were, on average, older than ours (mean age 52 months) and mostly males. Coronary artery lesions were present in 50% of the reported cases despite early treatment. Eladawy et al. [13] reported coronary dilatation in one patient and about 30% of refractory KD in a group of 7 children (100% males) with a mean age of 9.7 years, admitted with abdominal symptoms as a the main clinical manifestation. On the contrary, our data show that children with KD and gastrointestinal presentation are younger, without gender preponderance, and the CALs are present as ectasia in 16% and as aneurysms in 9.4% of cases.

Abdominal signs or symptoms are not part of the diagnostic criteria for KD, but sometimes may be the presenting or prominent complaint and can cause diagnostic delay, especially when diagnostic criteria are lacking during the febrile phase. As a matter of fact, in our cohort, patients with presenting abdominal manifestations more frequently underwent delayed treatment, probably due to atypical clinical presentation. Laboratory values (AST, sodium, neutrophiles, CRP) previously identified as risk factors for IVIG-resistance in the Japanese Risk Scoring Systems by Kobayashi, Egami and Sano [29, 30, 31] did not differ between the 2 groups in our study, except for PLT which were significantly higher, instead of lower, in patients with abdominal manifestations.

Our results could also suggest that the Asian risk score system, based on the Japanese population, is not well-performing in a predominantly Caucasian population, supporting previous data [32]. Lower serum albumin levels have previously been correlated with IVIG- unresponsiveness and CALs[33]. Our laboratory findings documented that during the acute phase of the disease serum albumin was significantly lower and PLT higher in patients with abdominal manifestations. Although the mechanism of hypoalbuminemia is multifactorial (decreased hepatic synthesis, increased urinary and intestinal protein loss), this finding may suggest increased intestinal inflammation and permeability since we did not find significant differences in albuminuria nor acute phase proteins (i.e. CRP) between subjects with and without gastrointestinal manifestations.

Our results show that clinical abdominal manifestations at the onset of KD in our population are a risk factor for severe coronary involvement, probably due to more severe and diffuse vasculitis involving the digestive tract.

Further prospective studies in non-Japanese populations are needed to support these findings.

Our study presents some limitations. First, being a retrospective multicenter study, not all of the patients had a complete set of all the data considered in the database. Second, as the echocardiographic evaluation was performed by the different participating institutions, there may be unknown biases in the study. Moreover, the Japanese classification used for defining CALs could underestimate abnormalities.

## Conclusion

Our findings show that presenting abdominal features in KD, regardless of their magnitude, identify a higher risk group for IVIG-resistance and coronary aneurysms in our cohort. Awareness must be focused especially in children younger than 6 months with high PLT and low serum albumin levels during the acute phase.

## Consent

The hospital ethics committee approved the publication of the data.

The authors state that there are no non-financial competing interests (political, personal, religious, ideological, academic, intellectual, commercial or any other) to declare in relation to this manuscript.

## Abbreviations

ALT: alanine aminotransferase
CALs: coronary artery lesions
CRP: C-reactive protein
ECG: electrocardiogram
ESR: erythrocyte sedimentation rate
2D Echo Doppler: two-dimensional color-flow Doppler echocardiography
gammaGT: gamma glutamyl transferase
Hb: hemoglobin
IVIG: intravenous immunoglobulin
L: lymphocites
N: neutrophils
Na: sodium
PLT: platelets
KD: Kawasaki disease
RBC: red blood cells
WBC: white blood cells
